# Effectiveness of P6 Stimulation and Transdermal Scopolamine Patch for the Reduction of Nausea and Vomiting During Caesarean Section Under Combined Spinal–Epidural Anesthesia: A Randomized Clinical Trial

**DOI:** 10.3390/jcm14072521

**Published:** 2025-04-07

**Authors:** Danielle Levin, Sarah Levin, Shaul Cohen

**Affiliations:** 1 Envision Physician Services and Department of Anesthesiology, Penn Medicine Princeton Medical Center, Plainsboro, NJ 08536l, USA; 2 Department of Anesthesiology and Perioperative Medicine, Rutgers-Robert Wood Johnson Medical School, RWJBarnabas Health System, New Brunswick, NJ 08901, USA; sjl220@scarletmail.rutgers.edu (S.L.); cohensh@rwjms.rutgers.edu (S.C.)

**Keywords:** antiemetics, caesarean section, combined spinal–epidural anesthesia, nausea, vomiting, P6 stimulation, scopolamine patch

## Abstract

**Background/Objectives:** Obstetric patients undergoing elective cesarean section (CS) with combined spinal–epidural (CSE) anesthesia often experience intraoperative nausea and vomiting (N&V). While prophylactic treatment with antiemetic drugs can be effective, it may also carry potential adverse effects for both the mother and the baby. To address this, we designed a randomized clinical trial to assess the effectiveness of transdermal scopolamine patches and electrical P6 stimulation as preventive measures for N&V in patients scheduled for elective CS under CSE anesthesia. **Methods:** Following the Institutional Review Board approval and informed consent, a total of 240 patients were randomly allocated into three groups: (1) transdermal scopolamine, (2) P6 stimulation (via a peripheral nerve stimulator), and (3) combined transdermal scopolamine and P6 stimulation, with 80 parturients in each group. The primary outcome was defined as the presence or absence of intraoperative nausea and vomiting during the procedure. **Results:** The incidences of intraoperative nausea and vomiting were similar across all three treatment groups, with no significant differences observed at any point during the surgery. Additionally, there were no notable differences in overall satisfaction with anesthetic care among the three study groups. **Conclusions:** These findings indicate that while both transcutaneous P6 acupoint stimulation and transdermal scopolamine are straightforward, safe, and effective methods, combining these two antiemetic strategies does not offer additional benefits in reducing nausea and vomiting. Nevertheless, both approaches may be particularly appealing to patients and obstetric anesthesiologists who prioritize treatments with fewer potential side effects.

## 1. Introduction

Nausea and vomiting are among the most common and distressing complications experienced during and after cesarean sections, particularly those performed under regional anesthesia. These symptoms not only cause significant discomfort for the patient but also pose additional risks that can complicate both the surgical procedure and the recovery process. Intraoperative nausea and vomiting may result in prolonged operative time, increased blood loss, and the potential for aspiration pneumonitis, particularly if vomiting occurs while the patient is in a supine position with an open airway. Furthermore, intraoperative nausea and vomiting can lead to unintentional surgical injury, especially in situations where the surgeon’s focus may be diverted or when a rapid intervention is required to manage the vomiting episode [1,2].

Despite the widespread use of intravenous antiemetics during cesarean sections, these medications are not always effective in preventing or treating nausea and vomiting. Traditional antiemetics, such as ondansetron, metoclopramide, and dexamethasone, often require repeat dosing and may be associated with a range of adverse effects. These include allergic reactions, cardiovascular instability (e.g., hypotension, tachycardia), gastrointestinal disturbances, and, in some cases, neurological or renal toxicity, especially in patients with comorbidities or those receiving multiple drugs [3,4,5,6]. Moreover, the effectiveness of these drugs can be diminished by their delayed onset of action or the pharmacokinetic properties of intravenous administration [7].

Given these limitations, there is increasing interest in alternative prophylactic and therapeutic strategies that can effectively prevent intraoperative nausea and vomiting while minimizing the risk of side effects. Among these, transdermal scopolamine has emerged as a promising option. Scopolamine is a centrally acting anticholinergic that works by blocking muscarinic receptors in the central nervous system, which helps prevent nausea and vomiting associated with vestibular and gastrointestinal stimuli. Several studies have demonstrated that transdermal scopolamine patches can effectively reduce the incidence of postoperative nausea and vomiting following elective cesarean sections [8,9]. In addition, scopolamine is considered non-teratogenic and has been found to be compatible with breastfeeding, making it an attractive option for postpartum patients [10]. However, while the efficacy of scopolamine in preventing postoperative nausea is well-established, there is a lack of clinical trials specifically examining its effectiveness in reducing nausea and vomiting during the intraoperative period itself, when patients are under the influence of regional anesthesia.

Another potential intervention with growing support is transcutaneous P6 acupoint stimulation, a non-pharmacological approach that targets the P6 acupoint (also known as the Nei Guan point) located on the anterior aspect of the forearm. This approach involves the application of low-frequency electrical pulses via a transcutaneous electrical nerve stimulation device. Research has shown that stimulation of the P6 acupoint can significantly reduce nausea and vomiting during surgery, especially in patients undergoing laparoscopic procedures and those undergoing cesarean deliveries [11]. The advantage of this technique lies in its non-invasive nature, lack of significant side effects, and ease of use. Transcutaneous acupoint stimulation works by modulating the vagal pathways and may promote the release of endogenous opioids, offering a physiological mechanism to mitigate nausea without relying on pharmaceuticals.

While these individual treatments—scopolamine and P6 acupoint stimulation—have demonstrated efficacy in reducing nausea and vomiting in various surgical settings, no studies have directly compared their intraoperative effectiveness in the context of cesarean sections. Furthermore, the combined effects of these two interventions remain largely unexplored. Given the complementary mechanisms of action—scopolamine as a systemic antiemetic and P6 stimulation as a localized, non-invasive treatment—there is potential for a synergistic effect when used in tandem.

Thus, the objective of this randomized clinical trial was to compare the incidence of intraoperative nausea and vomiting in patients undergoing cesarean sections under combined spinal–epidural anesthesia across three treatment groups: one receiving the transdermal scopolamine patch, one receiving transcutaneous P6 acupoint stimulation, and a third group receiving both interventions in combination. By examining the effects of these interventions in this setting, we aimed to provide evidence for more effective and safer management strategies for intraoperative nausea and vomiting during cesarean sections, contributing to better outcomes for patients undergoing this common surgical procedure.

## 2. Materials and Methods

### 2.1. Ethical Considerations

This randomized, controlled clinical trial was approved by the Institutional Review Board (IRB) at Rutgers-Robert Wood Johnson Medical School (protocol #PRO20160000234) and was registered on ClinicalTrials.gov (NCT02960113). The study was conducted in full compliance with the Consolidated Standards of Reporting Trials (CONSORT) guidelines, ensuring methodological transparency and rigorous reporting of results [12]. All participants provided written, informed consent prior to enrollment in the study, and their participation was entirely voluntary. Participants were informed of the potential risks and benefits of their involvement, including the specific treatments being investigated and the possible side effects. The study adhered to the ethical principles outlined in the Declaration of Helsinki, and patient confidentiality was maintained in accordance with the Health Insurance Portability and Accountability Act (HIPAA). The full trial protocol is available upon request from the first author of this manuscript. The clinical trial was concluded after all participants were successfully enrolled and completed follow-up assessments as per the study protocol.

### 2.2. Sample Size Justification

The sample size for this trial was determined using the Sealed Envelope online software (Version 12, Sealed Envelope Ltd., London, UK), designed for binary outcome superiority trials. Power calculations were performed to achieve a 90% statistical power with a two-tailed significance level of 5%, ensuring the ability to detect a meaningful difference in success rates between the treatment groups. The primary hypothesis was that the combination of transdermal scopolamine and transcutaneous P6 acupoint stimulation (group SP) would result in a 90% success rate in preventing intraoperative nausea and vomiting, compared to a 70% success rate in the single-treatment arms (group S and group P).

### 2.3. Study Population

Participants were selected from women scheduled for elective cesarean sections at a tertiary care teaching hospital in New Jersey, USA. The selection process began with a review of the obstetric operative calendar, which identified potential candidates based on their scheduled surgery. Inclusion criteria required participants to be 18–45 years of age, to have an ASA physical status class II, and to be scheduled for an elective cesarean section under combined spinal–epidural anesthesia.

Exclusion criteria included:

- History of placenta accreta or other placental abnormalities.
- Pregnancy-induced hypertension, preeclampsia, or eclampsia.
- Chronic medication use that could alter the perception of nausea and vomiting, such as long-term antiemetics, antidepressants, or antihistamines.
- Active systemic infections, including fever ≥ 38 °C, urinary tract infections, pneumonia, or otitis media.
- Presence of severe pre-existing gastrointestinal disorders (e.g., gastroparesis, active peptic ulcer disease).
- Known allergies or adverse reactions to any components of the study interventions (scopolamine, electrical stimulation).
- Severe psychiatric disorders or conditions preventing informed consent.
- Participation in another clinical trial within the previous 30 days.

The study began on 1 May 2016 and concluded on 1 December 2019. Eligible participants were identified during the preoperative assessment conducted by the anesthesia team. After the potential participants were informed about the study, they were invited to participate. Those who agreed to participate and met the inclusion criteria signed an informed consent form approved by the Institutional Review Board. Once consent was obtained, participants were randomly assigned to one of three groups (1:1:1) using a computer-generated randomization sequence:

1. Group S (transdermal scopolamine).
2. Group P (transcutaneous P6 acupoint stimulation).
3. Group SP (combination of transdermal scopolamine and transcutaneous P6 acupoint stimulation).

The randomization process was performed by the senior author, and the study was conducted in a single-center setting. Participants were not blinded to their treatment group, as per the nature of the interventions. Participants were informed that if they experienced nausea and/or vomiting during the procedure, they would have the option to request rescue antiemetic medications, with the final decision regarding administration made by the anesthesiologist.

### 2.4. Preoperative Protocol

In the preoperative holding area, all participants received standardized prophylactic medications to reduce the risk of aspiration and manage gastric acid secretion:

- A total of 30 mL of sodium citrate/citric acid was administered orally to neutralize gastric acid and reduce the potential for aspiration during anesthesia induction.
- A total of 20 mg of intravenous famotidine was administered to reduce gastric acid secretion and further decrease the risk of aspiration.

The participants were then prepared for anesthesia induction in the operating room. Using a sterile technique, the anesthesiologist identified the L3–L4 or L4–L5 interspace for combined spinal–epidural anesthesia. A 17G Tuohy needle was inserted using the loss-of-resistance-to-air technique. Once the correct location was confirmed, a 26G Gertie Marx spinal needle was used to administer a 2 mL mixture containing 0.5% bupivacaine, 20 μg of fentanyl, and 100 μg of epinephrine into the intrathecal space, ensuring adequate spinal anesthesia for the procedure. Following this, the spinal needle was removed, and a 17G epidural catheter was inserted 5 cm cephalad and secured with sterile dressing. Ice testing was performed to confirm adequate block height (T2–T4). In cases where additional anesthesia was required, 2% lidocaine with 5 μg/mL epinephrine was administered via the epidural catheter to achieve and maintain the desired sensory block.

Additionally, a bladder catheter was placed in all participants to ensure bladder decompression during the cesarean procedure.

### 2.5. Intraoperative Management

During the cesarean section, all participants were positioned supine with left uterine displacement. Continuous monitoring included an automated blood pressure cuff, electrocardiography, capnography, and pulse oximetry. Oxygen was administered via a nasal cannula at 4 L/min throughout the procedure. After delivery, 20 units of oxytocin were added to two 1 L bags of lactated Ringer’s solution and infused intravenously. No intravenous opioids were used during the procedure.

The blood pressure cuff was cycled every minute from the arrival to the operating room until the induction of the combined spinal epidural, followed by continuously for the next 10 min, and then every 3 min for the duration of the cesarean section. Hypertension was defined as a systolic blood pressure greater than 140 mm Hg at any point during the cesarean section, as shown in ref. [13], and hypotension was defined as a systolic blood pressure less than 90 mm Hg at any point during the cesarean section. In combination, 5 mg of ephedrine and 40 μg of IV phenylephrine were titrated to avoid and treat hypotension. Following the administration of IV phenylephrine and ephedrine, if blood pressure did not decrease below an SBP of 160 and/or a diastolic blood pressure of 110 within 3 min, the anesthesiologist had the option of administering 10 mg of IV esmolol to treat reactive hypertension. Excessive blood loss was defined as >1 L, estimated by the obstetrician based on the number of absorbent pads and the amount of suctioned blood. Severe hypoxemia was classified as oxygen saturation < 85% at any point during the procedure [14].

### 2.6. Interventions

#### 2.6.1. Group P (Transcutaneous P6 Acupoint Stimulation)

For participants in the P and SP groups, transcutaneous P6 acupoint stimulation was applied using a battery-powered peripheral nerve stimulator (EasyMed Instruments Co., Ltd., Foshan, China). Two disposable electrodes were placed over the right median nerve at the P6 acupoint, located approximately two fingerbreadths above the wrist joint crease, between the palmaris longus and flexor carpi radialis tendons. Stimulation began one hour before the administration of combined spinal–epidural anesthesia. The stimulator was set to deliver 1 pulse per second of direct current electrical stimulation at an intensity level gradually increased to the highest tolerated level. Stimulation continued throughout the surgical procedure until the participant arrived in the post-anesthesia care unit. The exact intensity of stimulation was titrated to the highest level that the participant could comfortably tolerate.

#### 2.6.2. Group S (Transdermal Scopolamine)

For participants in the S and SP groups, a 1.5 mg transdermal scopolamine patch (manufactured by Baxter Healthcare Corporation) was applied behind the ear one hour prior to the administration of spinal anesthesia. The patch remained in place throughout the surgical procedure and until the patient arrived in the post-anesthesia care unit. Participants were instructed that the patch could remain in place for up to three days if they wished to prolong its antiemetic effects after the procedure.

### 2.7. Rescue Antiemetic Therapy

In the event of intraoperative nausea or vomiting, participants were provided with rescue antiemetic therapy as per the discretion of the anesthesiologist. A combination of 10 mg of IV metoclopramide and/or 4 mg of IV ondansetron was administered based on the severity of symptoms and the participant’s preferences. Rescue antiemetic therapy was given promptly after the onset of nausea or vomiting to ensure optimal symptom control during the procedure.

### 2.8. Outcome Measurements

The primary outcome of the study was the presence or absence of intraoperative nausea and vomiting. Nausea and vomiting were assessed separately in four distinct phases of the operation:

1. From combined spinal–epidural anesthesia induction to uterine eversion.
2. From uterine eversion to uterine replacement.
3. From uterine replacement until the next 15 min.
4. From 15 min after uterine replacement until arrival at the PACU.

Secondary outcomes included:

- Patient satisfaction with the anesthetic care provided, measured using a numeric rating scale (0–10), where 0 = no satisfaction and 10 = complete satisfaction.
- The incidence of adverse events (e.g., hypersensitivity reactions, neuroleptic malignant syndrome, drowsiness, QT prolongation, etc.).
- Cardiac arrhythmias, as detected by continuous ECG monitoring during surgery.

### 2.9. Statistical Analysis

Descriptive statistics were used to summarize the distributions of continuous variables (mean ± standard deviation) and categorical variables (frequencies and percentages). Analysis of variance (ANOVA) was employed to compare the satisfaction scores and the severity of nausea at different time points across the three groups, while chi-square tests were used to evaluate the occurrence of nausea and vomiting overall and at each individual time point. Bonferroni correction was applied to adjust for multiple comparisons, ensuring control over type I error. The intent-to-treat analysis was performed for all primary and secondary outcomes to account for potential dropouts or non-compliance during the trial.

## 3. Results

### 3.1. Participant Flow

**Figure 1:**
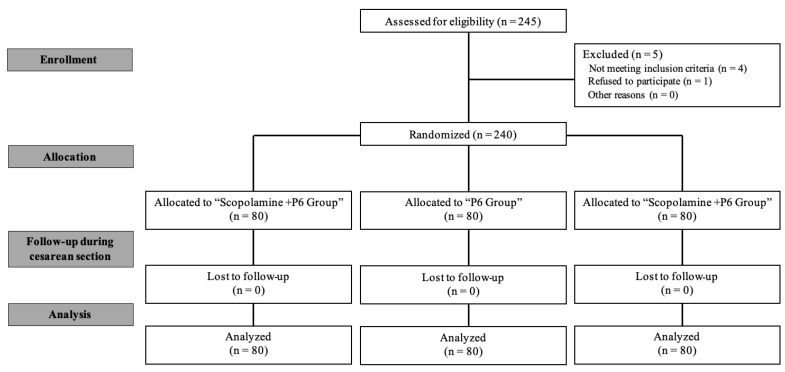
Flow diagram of the study as per the Consolidated Standards of Reporting Trials.

Of the 245 eligible patients (Figure 1), one woman withdrew from the study prior to surgery, and four patients had unsatisfactory epidural catheter placement for combined spinal–epidural anesthesia and therefore received general anesthesia instead. As a result, these patients were excluded from the final analysis. The remaining participants were randomly assigned to the intervention or control group. Analysis of variance (ANOVA) was used to compare age, gestational age, blood loss, and surgery duration across the three groups. Additionally, chi-square tests were employed to assess the incidence of female infants, twin infants, hypertension, hypotension, and hypoxia. Baseline characteristics, including demographic factors and procedural variables, were comparable between the groups, as shown in Table 1, suggesting that randomization was successful and that any observed differences in outcomes are unlikely to be confounded by baseline imbalances.

### 3.2. Primary Outcomes

The presence or absence of intraoperative nausea and vomiting was comparable across all three treatment groups, with no significant differences observed at any stage of the surgery (Table 2). The occurrence of intraoperative nausea and vomiting was monitored throughout the perioperative period, including during induction, intraoperatively, and in the immediate postoperative recovery phase. Statistical analysis confirmed that the rates of nausea and vomiting were similar across all groups, indicating that the interventions did not significantly impact the incidence of intraoperative nausea and vomiting.

### 3.3. Secondary Outcomes

There were no significant differences in overall treatment satisfaction among the three treatment groups (Table 3). Despite the variability in the specific interventions used across groups, the results suggest that none of the treatments significantly impacted patient satisfaction. These findings indicate that, from a patient perspective, the treatment methods tested were equally well-received.

Notably, no patient who received transcutaneous P6 acupoint stimulation reported discomfort from the device during the cesarean section. Some patients even noted that they “enjoyed the stimulator because it helped distract them from being awake during the procedure”, suggesting a positive subjective experience with the intervention.

Regarding safety, no patient required esmolol for hypertension treatment, and there were no adverse effects or complications associated with either the transcutaneous P6 acupoint stimulation or transdermal scopolamine. Additionally, all patients included in the analysis had adequate regional anesthesia for their cesarean section. A small number of patients (5 to 10 mL) required rescue epidural lidocaine with epinephrine to maintain adequate anesthesia; however, the exact number of patients who received this rescue medication was not documented.

These findings suggest that the interventions were not only well-tolerated but also did not result in significant adverse outcomes or complications, further supporting their safety and feasibility in this patient population.

## 4. Discussion

### 4.1. Cesarean Section and Anesthesia Overview

A cesarean section is a surgical intervention employed by obstetricians to facilitate the delivery of a baby through an incision made in the mother’s abdominal wall and uterus. This procedure is typically indicated when a vaginal delivery is not feasible, either due to complications with the pregnancy or maternal health or when vaginal delivery presents a risk to the well-being of the mother or fetus. While cesarean sections have become relatively common, their performance under anesthesia is a critical component of ensuring both maternal and fetal safety. The use of anesthesia not only ensures the comfort of the mother during the procedure but also minimizes pain and distress, allowing for a more controlled surgical environment. Anesthesia for cesarean sections is generally administered via one of three primary modalities: general anesthesia, spinal anesthesia, and epidural anesthesia. Each of these methods has distinct advantages and limitations, both in terms of safety and efficacy, impacting both the mother and the fetus in different ways [8,15].

### 4.2. General Anesthesia

General anesthesia involves the complete sedation of the patient, rendering them unconscious during the surgical procedure. The anesthetic agents used in this approach affect the entire body, inducing a state of deep sleep, complete muscle relaxation, and analgesia, which ensures that the surgical procedure can proceed without the patient being aware or feeling any pain. Although general anesthesia is a well-established and effective method for anesthesia administration, it is typically reserved for emergency cesarean sections or situations in which other forms of anesthesia are contraindicated. Under general anesthesia, the patient loses consciousness and experiences complete muscle relaxation, allowing the surgical team to perform the operation with full control. However, despite its effectiveness, general anesthesia carries an increased risk of maternal complications and mortality, compared to regional anesthesia methods. Studies have shown that, in elective procedures, avoiding general anesthesia whenever possible can significantly reduce maternal morbidity and mortality. As a result, it is less commonly used in planned cesarean sections, where alternative, safer methods of anesthesia are available [16].

### 4.3. Regional Anesthesia

In contrast to general anesthesia, regional anesthesia—comprising spinal anesthesia and epidural anesthesia—is the preferred method for elective cesarean sections. Regional anesthesia works by numbing the lower part of the body, typically through the injection of local anesthetic agents into the epidural or subarachnoid space surrounding the spinal cord. These anesthetics may include agents such as bupivacaine, ropivacaine, and fentanyl, which are selected based on their effectiveness in providing pain relief and minimizing side effects. In epidural anesthesia, a continuous infusion of anesthetic is administered to maintain the numbing effect, whereas in spinal anesthesia, a one-time, single-dose injection is used to achieve rapid onset and deeper anesthesia. Both methods allow the mother to remain awake and alert during the procedure, with the added benefit of significantly lower maternal morbidity, compared to general anesthesia. Furthermore, these regional techniques often lead to better neonatal outcomes, including higher Apgar scores at one and five minutes post-delivery, and they enable early bonding between the mother and infant. Despite these advantages, regional anesthesia is not without its challenges. In certain clinical situations, such as patients with coagulopathies or infections overlying the spine, regional anesthesia may be contraindicated. Additionally, it may result in side effects like nausea, vomiting, or residual pain post-surgery, which require management [17,18,19].

### 4.4. Medications for Nausea and Vomiting in Anesthesia

Nausea and vomiting represent common and distressing side effects following cesarean sections under regional anesthesia. These symptoms can arise due to the body’s response to anesthesia, the surgical procedure itself, or the hormonal changes associated with pregnancy. As such, effectively managing intra- and postoperative nausea and vomiting is crucial, not only to enhance patient comfort but also to expedite recovery. Numerous pharmacological agents have been developed to address these issues, each targeting different aspects of the body’s nausea and vomiting pathways. These agents include serotonin receptor antagonists such as ondansetron, dopamine receptor antagonists such as metoclopramide, corticosteroids such as dexamethasone, and antihistamines such as dimenhydrinate. Additionally, anticholinergic medications, such as scopolamine, have been widely used to prevent postoperative nausea and vomiting, especially in the perioperative period [20].

### 4.5. Anticholinergics—Scopolamine

Scopolamine, a commonly used anticholinergic agent, plays a key role in preventing nausea and vomiting after cesarean sections. Scopolamine works by blocking muscarinic receptors in the brain, which are primarily responsible for triggering the sensation of nausea. These receptors are concentrated in several key areas, including the chemoreceptor trigger zone and the vestibular system, which regulates balance and motion sickness. By antagonizing these receptors, scopolamine reduces the likelihood of nausea and vomiting following the surgical procedure. Scopolamine is typically administered as a transdermal patch, applied to the skin several hours before surgery, allowing for the slow release of the medication and its gradual action.

The use of scopolamine offers several benefits, including a lower risk of systemic side effects, compared to intravenous antiemetics. However, it is not without potential drawbacks. Common side effects of scopolamine include dry mouth, blurred vision, dizziness, and drowsiness. In rarer instances, more severe effects, such as confusion, memory disturbances, agitation, hallucinations, or delirium, may occur, particularly in elderly patients or those with cognitive impairments. Additionally, while scopolamine can cross the placenta, it is generally considered safe during pregnancy, as it is non-teratogenic (i.e., it does not cause birth defects) and is compatible with breastfeeding. Nevertheless, its use during pregnancy requires careful monitoring [9,21,22].

### 4.6. Non-Pharmacological Interventions

In addition to pharmacological treatments, non-pharmacological methods for managing nausea and vomiting have been investigated. One promising technique is P6 acupressure, which involves applying pressure to the Nei Guan (P6) acupoint, located on the inner forearm. A growing body of research suggests that stimulating the P6 acupoint can effectively reduce the incidence of postoperative nausea and vomiting. A meta-analysis of 40 trials involving over 4800 patients concluded that P6 acupressure is as effective as pharmaceutical interventions in mitigating nausea and vomiting. The non-pharmacological nature of acupressure means it is free from the side effects commonly associated with medications, and it allows for early maternal–infant bonding, as it does not interfere with the mother’s ability to hold and interact with her newborn following the surgery [23,24].

While P6 acupressure has demonstrated efficacy in some studies, its effectiveness remains debated. Some trials have failed to show significant differences between the acupressure and control groups, leading to inconclusive results. Additionally, electrical stimulation of the P6 acupoint has been explored as an alternative, with some suggesting that this approach may provide more consistent and reliable results, compared to manual pressure. Despite these promising avenues, there is still insufficient evidence to recommend P6 acupoint stimulation as a sole treatment for intraoperative nausea and vomiting during cesarean sections, especially in the context of regional anesthesia [11].

### 4.7. Study Objective

The current study sought to investigate the combined effects of a transdermal scopolamine patch and P6 acupressure on intraoperative nausea and vomiting during cesarean sections under combined spinal–epidural anesthesia. Over the past three decades, anesthesiologists at the study site have employed various antiemetic strategies, including intravenous metoclopramide, ondansetron, and transcutaneous P6 acupoint stimulation, to reduce nausea and vomiting in cesarean section patients. Given that transdermal scopolamine has been shown to effectively reduce postoperative nausea and vomiting, we hypothesized that combining it with P6 acupressure would provide an additive or synergistic effect, leading to improved outcomes.

Previous research by Kotelko et al. and Harnett et al. suggested that transdermal scopolamine patches reduce nausea and vomiting in patients undergoing cesarean delivery, particularly under spinal or epidural anesthesia. Kotelko et al. observed reductions in postoperative nausea and vomiting among patients who received preoperative scopolamine, while Harnett et al. reported that scopolamine reduced vomiting in the 6 to 24 h postoperative period following spinal anesthesia [8,15].

### 4.8. Study Results and Interpretation

Our study represents the first three-arm, parallel, randomized clinical trial to evaluate the combined effects of transdermal scopolamine and P6 acupressure on intraoperative nausea and vomiting in patients undergoing elective cesarean sections under combined spinal–epidural anesthesia. The incidences of nausea and vomiting were similar among parturients receiving scopolamine alone, P6 acupressure alone, or the combination of both treatments. These findings align with Levin et al.’s study, which demonstrated that transcutaneous P6 acupoint stimulation could reduce intraoperative nausea and vomiting. However, it remains unclear whether the addition of scopolamine or other antiemetics to P6 acupressure would provide additional benefits [11].

The underlying mechanism of P6 acupoint stimulation is still not fully understood, but it is based on the concept of restoring balance within the body’s energy pathways (meridians). By stimulating the P6 acupoint, it is believed that energy flow is normalized, potentially reducing nausea and vomiting without inducing adverse effects [25,26,27]. While some trials have shown positive results, other studies, including those by Woodward et al. and El-Deeb et al. [27,28], have questioned the overall effectiveness of non-invasive acupressure methods in reducing intraoperative nausea and vomiting.

### 4.9. Limitations of the Study

A major limitation of our study is the lack of a control group and the absence of a completely blinded design, which may affect the generalizability of our findings. Furthermore, the anesthesiologist responsible for assessing the need for rescue antiemetics was aware of the group assignments, which could introduce bias into the decision-making process. Additionally, patients provided subjective evaluations of the quality of care received, and given the inherent subjectivity of this assessment, it may introduce bias. To confirm these results and explore the full potential of combined antiemetic therapies, further studies with larger sample sizes, randomized designs, and blinded assessments are needed.

The sample size was calculated based on a ‘success rate in preventing nausea and vomiting’, which may not fully align with the primary outcome, defined as the ‘presence or absence of intraoperative nausea and vomiting’. While recalculating the statistical power with this definition was not possible at the time of writing, future studies could benefit from recalculating the sample size based on the primary outcome for a more precise statistical analysis.

## 5. Conclusions

Our results showed that the incidences of intraoperative nausea and vomiting during cesarean sections under combined spinal–epidural anesthesia were similar among parturients who received transdermal scopolamine alone, transcutaneous P6 acupoint stimulation alone, or both treatments combined. Additionally, satisfaction with treatment was comparable across all three groups. These findings suggest that while transcutaneous P6 acupoint stimulation and transdermal scopolamine are both simple, safe, and effective approaches, the combination of these two antiemetic strategies does not appear to provide an additional benefit in reducing nausea and vomiting. We anticipate that future prospective randomized clinical trials will further investigate potential combinations to reduce the incidence of intraoperative nausea and vomiting during cesarean sections.

## Figures and Tables

**Table 1:** Patient and procedural characteristics.

Characteristics	Scopolamine Group (n = 80)	P6 Group (n = 80)	Scopolamine + P6 Group (n = 80)
Age (years)	32.8 ± 6.2	31.3 ± 5.3	32.1 ± 4.9
Gestational age (weeks)	38.4 ± 1.7	38.5 ± 1.4	38.7 ± 0.8
Female infant, n (%)	29 (43%)	33(49%)	31(49%)
Twin infants, n (%)	1(1%)	1(2%)	2 (3%)
Hypertension, n (%)	43 (54)	50 (63)	47 (59)
Hypotension, n (%)	38 (47)	40(50)	35 (44)
Hypoxia, n (%)	0 (0)	0 (0)	0 (0)
Blood Loss (mL)	818.8 ± 215.2	764.2 ± 128.5	820.9 ± 205.8
Duration of surgery (min)	63.2 ± 17.5	60.1 ± 16.9	65.5 ± 16.7

Continuous variables were expressed as mean ± standard deviation. Categorical variables were expressed as a number (percentage).

**Table 2:** Comparison of nausea and vomiting rates during CS with respect to the group allocation.

N&V	Scopolamine Group (n = 80)	P6 Group (n = 80)	Scopolamine + P6 Group (n = 80)	*p*-Value
Nausea, n (%)				
Overall	40 (50%)	39 (49%)	46 (58%)	0.49
Stage 1	27 (34%)	27 (34%)	29 (36%)	0.70
Stage 2	13 (16%)	11 (14%)	14 (18%)	0.51
Stage 3	0 (0%)	0 (0%)	0 (0%)	1.00
Stage 4	0 (0%)	0 (0%)	0 (0%)	1.00
Vomiting, n (%)				
Overall	20 (25%)	15 (19%)	20 (25%)	0.56
Stage 1	9 (10%)	6 (8%)	9 (11%)	0.72
Stage 2	10 (13%)	15 (19%)	12 (15%)	0.55
Stage 3	1 (1%)	1 (1%)	4 (5%)	0.25
Stage 4	24 (30%)	25 (31%)	31 (29%)	0.45

Categorical variables were expressed as a number (percentage). Stage 1 = from the administration of CSE and until eversion of the uterus. Stage 2 = after eversion of the uterus and until the replacement of the uterus. Stage 3 = after replacement of the uterus and to the next 15 min. Stage 4 = the rest of the time until arrival at PACU.

**Table 3:** Comparison of satisfaction scores with respect to the group allocation.

Satisfaction	Scopolamine Group (n = 80)	P6 Group (n = 80)	Scopolamine + P6 Group (n = 80)	*p*-Value
Overall care	9.59 ± 0.86	9.82 ± 0.55	9.83 ± 0.63	0.05

Continuous variables were expressed as mean ± standard deviation.

## Data Availability

The raw data supporting the conclusions of this article will be made available by the authors upon request.
